# Pilot study for bladder cancer detection with volatile organic compounds using ion mobility spectrometry: a novel urine-based approach

**DOI:** 10.1007/s00345-024-05047-5

**Published:** 2024-05-25

**Authors:** Hendrik Heers, Josef Maximilian Gut, Rainer Hofmann, Luka Flegar, Marcus Derigs, Johannes Huber, Joerg Ingo Baumbach, Andreas Rembert Koczulla, Tobias Boeselt

**Affiliations:** 1 Department of Urology, Philipps-Universität Marburg, Baldingerstraße, 35033 Marburg, Germany; 2 Department of General and Visceral Surgery, München Klinik Neuperlach, Oskar-Maria-Graf-Ring 51, 81737 Munich, Germany; 3 Department Bio- and Chemical Engineering, Technical University Dortmund, Emil-Figge-Straße 66, 44227 Dortmund, Germany; 4 Department of Pulmonology, Philipps-Universität Marburg, Baldingerstraße, 35033 Marburg, Germany; 5 Department of Pulmonology, Schön-Klinik Berchtesgadener Land, Malterhöh 1, 83471 Schönau, Germany

**Keywords:** Bladder cancer, Volatile organic compounds, Ion mobility spectrometry, Biomarker

## Abstract

**Purpose:**

Despite many efforts, no reliable urinary marker system has so far shown the potential to substitute cystoscopy. Measuring volatile organic compounds (VOCs) from urine is a promising alternative. VOCs are metabolic products which can be measured from the headspace of urine samples. Previous studies confirmed that the urine of bladder tumor patients has a different VOC profile than healthy controls. In this pilot study, the feasibility of discriminating VOCs from urine of bladder cancer patients from that of healthy control subjects was investigated. Aim of this study was to investigate whether VOC-based diagnosis of bladder cancer from urine samples is feasible using multicapillary column ion mobility spectrometry (MCC/IMS) and to identify potential molecular correlates to the relevant analytes.

**Methods:**

Headspace measurements of urine samples of 30 patients with confirmed transitional cell carcinoma (TCC) and 30 healthy controls were performed using MCC/IMS. In the results of the measurements, peaks showing significant differences between both groups were identified and implemented into a decision tree with respect to achieve group separation. Molecular correlates were predicted using a pre-defined dataset.

**Results:**

Eight peaks with significantly differing intensity were identified, 5 of which were highly significant. Using a six-step decision tree, MCC/IMS showed a sensitivity of 90% and specificity of 100% in group separation.

**Conclusion:**

VOC-based detection of bladder cancer is feasible. MCC/IMS is a suitable method for urine-based diagnosis and should be further validated. The molecular characteristics and metabolic background of the analytes require further workup.

**Supplementary Information:**

The online version contains supplementary material available at 10.1007/s00345-024-05047-5.

## Introduction

Bladder cancer has been showing a slow rise in incidence over the past years. In Germany, there were a total of 31,040 cases in 2018; one third of those were non-invasive pTa tumours [1]. 80% of bladder cancer patients present with haematuria which accounts for the fact that most patients are diagnosed at an early stage [2]. Hence, all patients with visible haematuria should undergo a cystoscopy which will be positive in 20% of cases. Cystoscopy is also required during the follow-up of non-muscle invasive bladder cancer as there is a significant risk of recurrence.

Many attempts have been made to evade cystoscopy as it is not only unpopular with patients due to its invasive and uncomfortable nature but also time-consuming and expensive. So far, no urine-based biomarker system other than cytology has successfully been implemented into clinical routine and guidelines [3]. The measurement of volatile organic compounds (VOC) could be a promising alternative to conventional urinary biomarkers. VOCs are metabolic products which are emitted via breath, urine, and other body secretions. Many diseases lead to characteristic alterations in VOC profiles which can be used for diagnostic purposes. VOC detection has been shown to be effective in the diagnosis of a range of pulmonary, neurological, and malignant disease from breath as well as inflammatory bowel disease from stool [4–10]. Our group previously reported a pilot study on the VOC-based detection of bladder cancer from the headspace of urine samples using an electronic nose [11]. Electronic noses use pattern recognition to allocate gas samples to groups. Measurements are fast and can be conducted at the bedside. However, those systems do not measure individual molecular components of the gas sample. Therefore, an identification of substances as potential targeted biomarkers is not possible. Ion mobility spectrometry (IMS) is a semi-quantitative method which can differentiate between groups and enables the identification of individual components of a mixed gas sample (using a reference dataset).

The aim of this study was to establish the method of headspace measurements independent from ambient air for measuring and distinguishing urine samples and to create specific VOC profiles using IMS as a novel non-invasive, cost-effective, and precise method to detect bladder cancer.

The primary objective was to evaluate whether IMS could detect differences between the smell print of urine samples of bladder cancer patients and healthy controls. Secondary objectives were to investigate whether IMS was more accurate than measurements using electronic noses and to identify individual molecular substances specific to bladder cancer which could potentially be used as biomarkers in the future.

## Materials and methods

### Patients and sample storage

Fourty-two patients with cystoscopically confirmed bladder tumors referred for TUR-BT were recruited on the day of their pre-assessment. Patients with urinary tract infection on urine dipstick, indwelling bladder catheters or ureteric stents, and patients with known other malignancies were excluded. Twelve patients were excluded as no malignancy was found on histopathological workup. Thirty individuals with no known disease of the urinary tract (including urinary tract infections) were recruited as healthy controls. Samples of first void morning urine were collected for analysis and stored in 2 ml vials at −20 °C until measurement.

Ethics approval for this study was given by the local ethics committee (Az 131/14). All participants were informed about the study and handed an information sheet; written consent was obtained.

### Headspace measurements

Samples were thawed at room temperature and then vortexed and heated to 37 °C in a water bath for 10 min before being transferred into a sealable vial that could be connected to the tubing system of the IMS. We used a BioScout IMS (B and S Analytik GmbH, Dortmund, Germany) combined with an up-streamed multicapillary column (MCC, type OV-5, Multichrom Ltd, Novosibirsk, Russia) to pre-grade volatile analytes. Gaseous material arising from the heated urine samples was exposed and sucked into the IMS for measurement. A standardised flushing procedure to clean the sensors was performed according to the manufacturer’s recommendations after each measurement.

### Analysis of MCC-IMS data

Raw data from IMS measurements was analyzed using the software Visual Now 2.2 (B and S Analytik GmbH, Dortmund, Germany). The electronic signals caused by single ion impact were visualized as peaks, the height of which being characterized by ion concentration and its position with regard to drift time and retention time. Hence, every sample running through the spectrometer created a unique set of peaks. Based on the database 20160426_SubstanzDbNIST (B and S Analytik GmbH, Dortmund, Germany), the peaks were compared to database-derived characteristics of known substances using MIMA, a software for VOC detection. Rapid Miner 7.5 (Rapid Miner GmbH, Boston, MA, USA) was used to generate a multi-level decision tree to differentiate between groups of samples.

The team in charge of IMS data analysis was blinded to the results of the pathology reports and vice versa.

### Statistics and data analysis

All IMS analyses were calculated with SPSS 22 (IBM SPSS Statistics, Version 22.0. Armonk, New York, USA) and Prism 5.03 (GraphPad Software, Inc., La Jolla, USA). Mann–Whitney-U-Test for unpaired samples was used to compare between two groups while for within-group comparisons, a Wilcoxon rank-sum Test for paired samples was performed. For comparison of measurements from several time-points, Friedman’s test for continuous and Fisher’s exact test for categorical variables were performed. All tests were two-sided (p < 0.05 was considered to be significant). For independent samples, the Kruskal–Wallis test was used. For patient and tumor characteristics, categorical variables were evaluated as counts and percentages, and chi-square test was used; continuous variables were evaluated by using mean ± standard deviation; parametric variables were evaluated with *t*-test.

## Results

The demographics of study participants are summarized in Table 1.

**Table 1:** Patient demographics

	Tumor (n = 30)	Control (n = 30)	p value
Mean age ± SD (range) [years]	71.3 ± 10.0 (49–86)	56.4 ± 17.6 (21–85)	< 0.001
Sex (male:female)	24:6	21:9	0.4
History of smoking, no. of patients (%)	15 (50%)	9 (30%)	0.1
Microscopic hematuria, no. of patients (%)	18 (60%)	7 (23.3%)	0.004

**SD** Standard deviation

Histopathology confirmed urothelial carcinoma in 30 patients from the tumor group with a representative range of tumor stages. Tumor characteristics are shown in Table 2.

**Table 2:** Tumor characteristics

	n (%)
**Grade (WHO 2016)**	
Low grade	14 (46.7%)
High grade	16 (53.3%)
**Stage**	
pTa	17 (56.7%)
pT1	4 (13.3%)
CIS	1 (3.3%)
pT1 + CIS	2 (6.7%)
≥ pT2	5 (16.7%)
pT2 + CIS	1 (3.3%)
**Focality**	
Monofocality	21 (70%)
Multifocality	9 (30%)
**Maximum tumor diameter**	
< 3 cm	17 (56.7%)
≥ 3 cm	13 (43.3%)
**Tumor status**	
Primary	19 (63.3%)
Recurrent	11 (36.7%)

A total of 82 peaks representing individual molecular components of the gas samples were identified. Of those, 8 peaks showed significantly different levels of signal intensity between groups with p < 0.01 and 5 peaks were highly significant with p < 0.001.

**Fig. 1:**
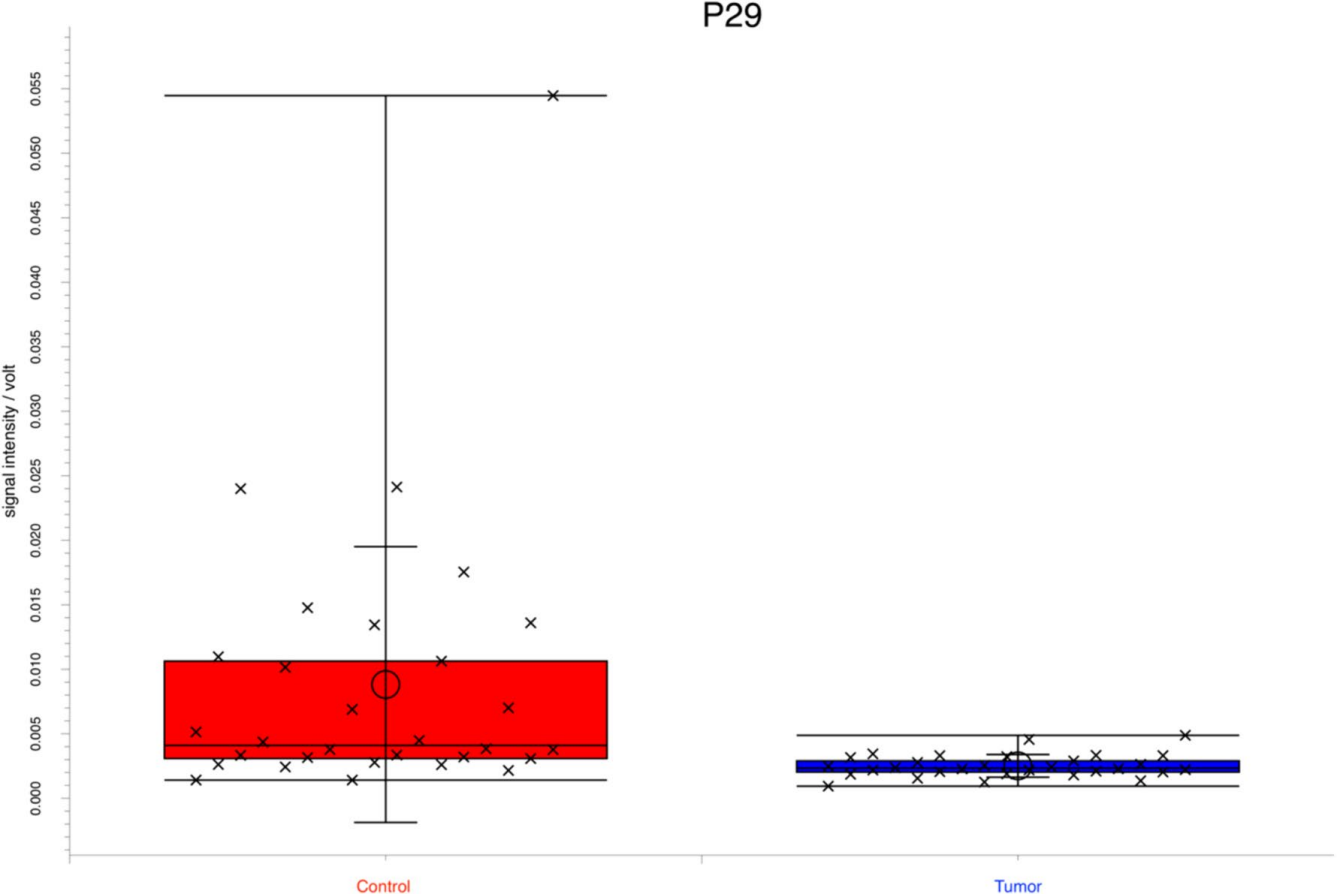
Box whisker plot for peak P29 with a significantly higher signal intensity in the control group (red) compared with the tumor group (blue). In accordance to the reference database, the corresponding substance was predicted to be benzaldehyde or benzofurane

As an example, Fig. 1 shows the box whisker plot for peak P29 after Bonferroni correction.

**Fig. 2:**
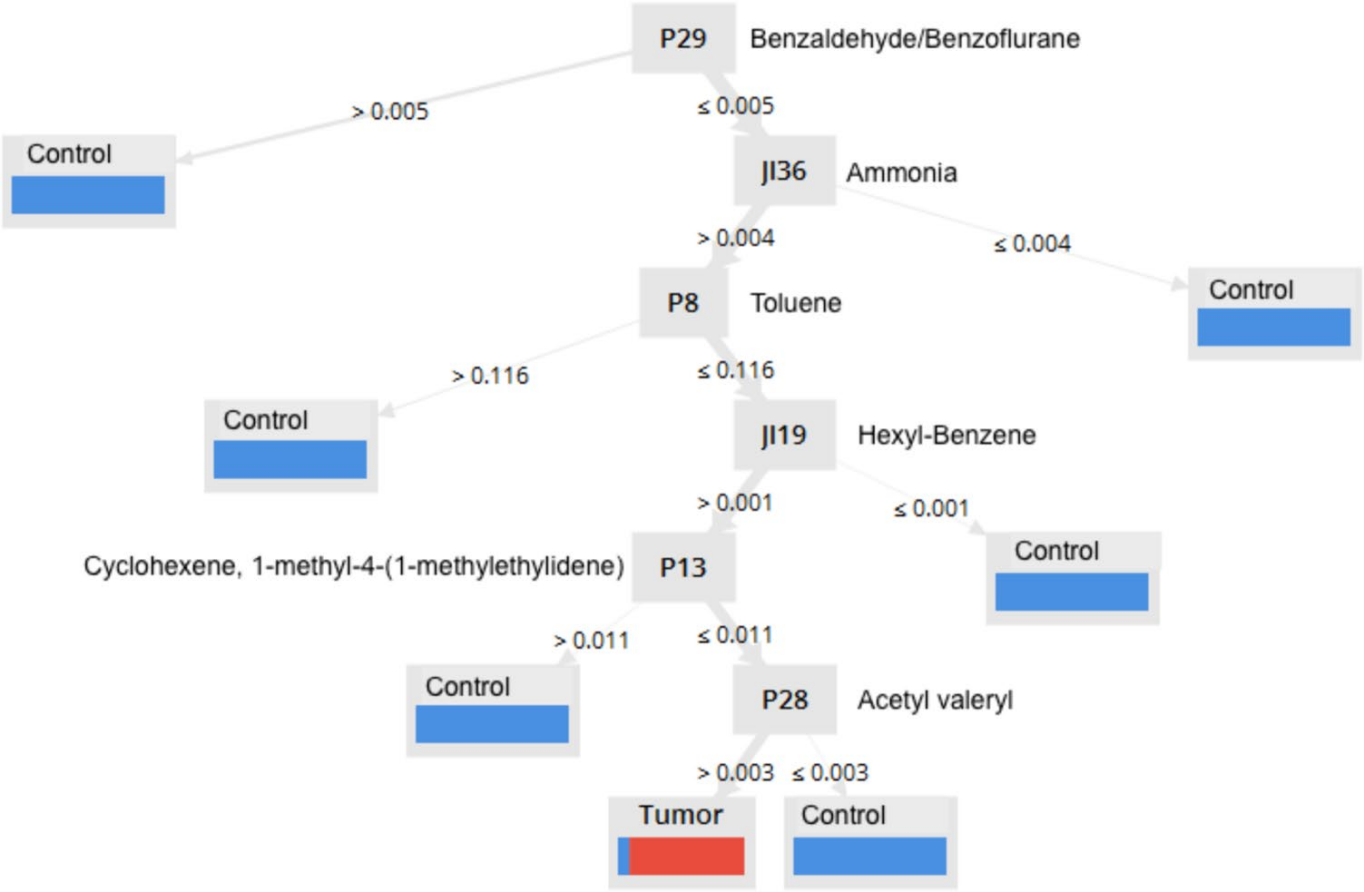
Decision tree with six steps based on the most significant peaks and the presumed molecular correlates

In order to differentiate between tumor and control samples, a decision tree based on significant peaks was generated using six steps (Fig. 2).

This resulted in a sensitivity of 90% and a specificity of 100%. Further statistics of the decision tree are shown in Table 3.

**Table 3:** Statistical characteristics of the decision tree

Statistical analysis	Control	Tumor	Total	
Prediction healthy control	27	0	27	
Prediction tumor	3	30	33	
Total	30	30	60	
Sensitivity	90%			
Specificity	100%			
PPV	100%		False positive	0%
NPV	91%		False negative	9%
AUC	95			
95% confidence interval				
Sensitivity	101	79		
Specificity	100	100		
Prevalence = AUC	101	89		

The peaks which were used in this model were compared with the aforementioned database in order to identify potential molecular correlates based on previous IMS experiences followed by in-depth molecular characterization of analytes. The following possible correlations were established: P29: Benzylaldehyde (dimer) or benzofurane; JI36: ammonia; P8: toluol; JI19: hexylbenzene; P13: cyclohexene 1-methyl-4-(1-methylethylidene); P28: acetyl valeryl.

No significant differences in measurement patterns could be identified between high grade and low grade tumors or for different T stages or for recurrent versus primary disease. Results for smokers and non-smokers did not differ. The presence of microscopic hematuria did not result in significantly different results either.

## Discussion

In this pilot study, small cohorts of confirmed bladder cancer patients and healthy controls were compared. Using MCC/IMS, it could be shown that the two groups have different VOC profiles and the peaks relevant for group separation were identified.

Using a decision tree-based model, IMS could separate between urine samples of bladder cancer patients and healthy controls with a sensitivity of 90% and specificity of 100%. Potential molecular correlates to the most relevant individual gaseous analytes were suggested.

Little research has been done so far on VOC-based diagnosis of bladder cancer. While only using a comparatively small sample size and a highly standardized setting, our study provides further evidence that bladder cancer diagnosis based on VOC analysis is feasible in principle. They confirm our previous findings with the electronic nose Cyranose 320^™^ which showed a sensitivity of 93.3% and specificity of 87.7% [11].

Other groups found similar results with different VOC-based setups for bladder cancer diagnosis [12–16]. While those high values for sensitivity and specificity are promising and warrant further investigations of the method, the potential influence of confounders such as urinary tract infections or hematuria remains to be established in larger studies. At present, we did not find any differences in the measurement patterns of patients with or without microscopic hematuria. However, this might be an effect of the small sample size in this pilot study.

A follow-up study validating the method in patients presenting for hematuria workup is currently underway at our center. Another aspect to be looked at in future research is whether VOCs can help to differentiate between high and low grade tumors or T stages. In this limited data set, we did not find significant differences.

In comparison to VOC-based methods, the only urine-based diagnostic method recommended for bladder cancer by various guidelines — nurine cytology — has a sensitivity of 34% and specificity of 99% [17]. A plenitude of urine-based marker systems has been introduced over the years but none was convincing enough to be implemented into clinical routine. The most relevant were summarized in a systematic review by Ng in 2021 [3]. NMP22 demonstrated a sensitivity of 52–59% and a specificity of 87–89%. The BTA Stat test performed at a sensitivity of 57–82% and specificity of 68–93%, and FISH-based UroVysion showed a sensitivity of 69–87% and specificity of 89–96%. Multigene panels and miRNA showed potentially better results depending on the approach but are more expensive and time-consuming. Hence, pattern recognition based on VOCs which also uses a multi-target approach seems to be a more sensible approach than conventional biomarkers. Cystoscopy itself, which remains the golden standard, was found to have a sensitivity of 62–84% and specificity of 43–93%. When using photodynamic diagnostics, this improved the sensitivity to 82–97% at a specificity of 35–98% [18]. Keeping that in mind, a non-invasive option sparing patients from cystoscopy seems not too far away.

Within the range of VOC detection methods, MCC/IMS represents a compromise between rapid and easy to perform approaches solely based on pattern recognition such as electronic noses and most accurate but slow and expensive methods allowing for single analyte characterization such as gas chromatography/mass spectrometry (GC/MS). One advantage is that it allows to obtain time serial measurements enabling insight into the dynamics of VOC release. The current IMS applications available require manual analysis, but for different indications, bedside variations have already been introduced since 2017 [19].

A strength of this study is its simple design with direct comparison of tumor patients and benign control aiming for optimal group separation. The patient cohort was representative for bladder cancer patients in a real-world setting. One of its limitations clearly is the comparatively small sample size.

The mean age between patients in the two groups differed significantly. Given our experience from previous studies, it is unlikely but conceivable that this factor would influence the VOC profile.

The findings have yet to be validated in a cohort at risk such as patients with visible hematuria undergoing a workup for bladder cancer. This is being investigated in the aforementioned follow-up study. It remains to be seen whether the very promising sensitivity and specificity can be reproduced in a non-filtered cohort.

Last but not least, the analytes that we suspect behind the most significant measurement peaks may or may not be correctly predicted and require further analysis with GC/MS. The underlying metabolic pathways are still unclear.

These should be the next steps in implementing the VOC-based approach. Nevertheless, there is evidence that single analyte marker systems for bladder cancer are inferior to multi-target setups. Thus, pattern recognition may prove to be more sensible than developing a targeted measurement system for individual analytes identified by MCC/IMS or GC/MS.

MCC/IMS as a technology is promising and reliable but so far, it is solely an exploratory method which requires expert data handling with time-consuming manual peak picking, hence it is not a good candidate for clinical routine in the current form. However, there is already work being done in order to implement artificial intelligence algorithms to significantly simplify data analysis.

With regard to a future transition of VOC detection from research to a clinical routine laboratory, MCC/IMS and GC/MS findings may also help to identify the relevant panel of molecular components that are responsible for the smell print of bladder cancer in urine. Those components could then be included in a purpose-designed electronic nose which would enable rapid bedside testing. This approach of modified “nose chips” is already being practiced by manufacturing companies in the field of airport and military security.

Whether VOC-based methods have a role in follow-up for NMIBC and in the detection of upper urinary tract urothelial cancer is yet to be determined.

In conclusion, the detection of volatile organic compounds is a promising approach for reliable non-invasive diagnosis and potentially for the follow-up of bladder tumors. While the current clinical significance of the method is limited, it warrants further validation studies with larger sample sizes.

## Supplementary Information

Below is the link to the electronic supplementary material.

Supplementary file1 (XLSX 33 KB)

## Data Availability

All relevant data supporting the findings of this study are available within the paper. Raw measurement data from MCC/IMS of individual samples are available from the corresponding author upon reasonable request. Data are located in controlled access data storage at Philipps-Universität Marburg.
